# Histone modifications and their role in epigenetics of atopy and allergic diseases

**DOI:** 10.1186/s13223-018-0259-4

**Published:** 2018-05-23

**Authors:** Bilal Alaskhar Alhamwe, Razi Khalaila, Johanna Wolf, Verena von Bülow, Hani Harb, Fahd Alhamdan, Charles S. Hii, Susan L. Prescott, Antonio Ferrante, Harald Renz, Holger Garn, Daniel P. Potaczek

**Affiliations:** 1 Institute of Laboratory Medicine and Pathobiochemistry, Molecular Diagnostics, Philipps University Marburg, Hans-Meerwein-Straße 3, 35043 Marburg, Germany; 2 inVIVO Planetary Health, Group of the Worldwide Universities Network (WUN), New York, NJ USA; 3 Department of Immunopathology, SA Pathology, Women and Children’s Hospital Campus, North Adelaide, SA Australia; 4 Robinson Research Institute, School of Medicine and School of Biological Science, University of Adelaide, Adelaide, SA Australia; 5 School of Paediatrics and Child Health, University of Western Australia, Perth, WA Australia; 6 German Center for Lung Research (DZL), Gießen, Germany; 7 John Paul II Hospital, Krakow, Poland; 8 Present Address: Boston Children’s Hospital, Harvard Medical School, Boston, MA USA

**Keywords:** Allergy, Asthma, Atopy, Epigenetic (-s), Histone acetylation, Histone methylation, Histone modification (-s), Histone phosphorylation, Inflammation, Immunity

## Abstract

This review covers basic aspects of histone modification and the role of posttranslational histone modifications in the development of allergic diseases, including the immune mechanisms underlying this development. Together with DNA methylation, histone modifications (including histone acetylation, methylation, phosphorylation, ubiquitination, etc.) represent the classical epigenetic mechanisms. However, much less attention has been given to histone modifications than to DNA methylation in the context of allergy. A systematic review of the literature was undertaken to provide an unbiased and comprehensive update on the involvement of histone modifications in allergy and the mechanisms underlying this development. In addition to covering the growing interest in the contribution of histone modifications in regulating the development of allergic diseases, this review summarizes some of the evidence supporting this contribution. There are at least two levels at which the role of histone modifications is manifested. One is the regulation of cells that contribute to the allergic inflammation (T cells and macrophages) and those that participate in airway remodeling [(myo-) fibroblasts]. The other is the direct association between histone modifications and allergic phenotypes. Inhibitors of histone-modifying enzymes may potentially be used as anti-allergic drugs. Furthermore, epigenetic patterns may provide novel tools in the diagnosis of allergic disorders.

## Background

In the last few decades, there has been a substantial increase in the prevalence of allergic diseases in the industrialized countries [1–3]. Since this change could not be explained by a rather stable population genetic profile [2–4], increased exposure to harmful and reduced exposure to protective epigenetically-mediated environmental factors have been considered, at least in part, as a possible explanation for this epidemiological phenomenon [5–9]. While DNA methylation has been extensively studied as the epigenetic mechanism involved in the etiopathogenesis of allergic disorders, posttranslational histone modifications, another important classical epigenetic mechanism, have not been as widely investigated and discussed because it is not considered as important as DNA methylation [5–7, 10]. The review firstly describes the (bio-) chemical basics of epigenetic histone modifications. This is followed by an assessment of recent evidence that supports a role for histone modifications in the epigenetic regulation of the pathogenesis of allergy and related disorders, together with a description of the underlying cellular and molecular mechanisms.

## Main text

### Histone modifications: the basics

Similarly to DNA methylation, posttranslational histone modifications do not affect DNA nucleotide sequence but can modify its availability to the transcriptional machinery. Although histone modifications play also other roles, such as histone phosphorylation, best known for its contribution to DNA repair in response to cell damage, this review deals primarily with general mechanisms of histone modifications in the context of their role in epigenetic modulation of gene expression. Several types of histone modifications are known, amongst which acetylation, methylation, phosphorylation, and ubiquitination are the best studied and most important in terms of the regulation of chromatin structure and (transcriptional) activity [11–15]. In general, histone modifications are catalyzed by specific enzymes that act, predominantly, but not exclusively (e.g. some types of histone phosphorylation), at the histone *N*-terminal tails involving amino acids such as lysine or arginine as well as serine, threonine, tyrosine, etc. Histone acetylation usually leads to higher gene expression. This may not always be the case for histone H4 [16–18]. Histone methylation in turn has either transcriptionally permissive or repressive character, depending on the location of targeted amino acid residues in the histone tail and/or the number of modifying (e.g. methyl) groups added [5, 6, 14, 15, 19, 20]. Table 1 summarizes the various forms of histone modifications appearing in this review along with their effects on gene transcriptional activity.

**Table 1:** List of histone modifications appearing in this review along with their effects on the transcriptional activity

Modification and site	Abbreviation	Effect on transcription^a^
Histone acetylation		
Histone 3 panacetylation	H3ac	Activating/permissive
Histone 4 panacetylation	H4ac	Activating/permissive^b^
Histone 3 lysine 4 acetylation	H3K4ac	Activating/permissive
Histone 3 lysine 9 acetylation	H3K9ac	Activating/permissive
Histone 3 lysine 14 acetylation	H3K14ac	Activating/permissive
Histone 3 lysine 18 acetylation	H3K18ac	Activating/permissive
Histone 3 lysine 27 acetylation	H3K27ac	Activating/permissive
Histone 4 lysine 16 acetylation	H4K16ac	Activating/permissive
Histone methylation		
Histone 3 lysine 4 methylation	H3K4me1	Activating/permissive
Histone 3 lysine 4 dimethylation	H3K4me2	Activating/permissive
Histone 3 lysine 4 trimethylation	H3K4me3	Activating/permissive
Histone 3 lysine 9 dimethylation	H3K9me2	Repressive
Histone 3 lysine 9 trimethylation	H3K9me3	Repressive
Histone 3 lysine 27 trimethylation	H3K27me3	Repressive
Histone 3 lysine 36 trimethylation	H3K36me3	Activating/permissive
Histone 3 lysine 79 methylation	H3K79me1	Activating/permissive
Histone phosphorylation		
Histone 3 serine 10 phosphorylation	H3S10ph	Activating/permissive
Histone ubiquitination		
Histone 2A ubiquitination	H2Aub	Repressive
Histone 2B ubiquitination	H2Bub	Activating/permissive

^a^According to the cells signaling technology webpage [100] and/or other sources referenced in the “Main text” and/or Tables 2 and 3 of this review

^b^See also “Histone modifications: the basics”

**Table 2:** Studies on the role of histone modifications in allergic diseases meeting the primary selection criterion

Study	Major epigenetic aims	Major epigenetic results
Harb et al. [84]	Analysis of the association between prenatal fish oil exposure (maternal fish oil intake) and CB CD4+ T-cell H3ac and H4ac levels at promoters of *PRKCZ* and several other important T-cell genes (placebo n = 34; fish oil n = 36)	Significantly higher H3ac levels at the *PRKCZ* and *IFNG*, and lower H3/H4ac levels at the *IL13* and *TBX21* were observed in CB CD4+ T-cells obtained from newborns of mothers supplemented with fish oil during pregnancy compared to newborns of placebo-treated mothers. The infants born from the fish oil-supplemented mothers were at the lower risk of developing allergic diseases [81, 82]
Harb et al. [83]	Comparison of H3ac and H4ac levels at gene promoters of Th1, Th2, Th17, and Treg cells between CB CD4+ T-cells obtained from neonates with either high (n = 12) or low (n = 11) maternal serum folate levels estimated during the last trimester selected form a larger cohort based on conventional extremes of exposure design	Significantly higher *GATA3* promoter H3ac and H4ac levels were observed in the high folate group. Significantly higher *IL9* promoter H4ac levels in high folate arm (and a tendency towards a similar association for H3ac) were also found. A tendency towards lower *IFNG* promoter H4ac was observed in high folate group. Statistical analyses included adjustment for CB serum vitamin D levels
Stefanowicz et al. [66]	Comparison of global and gene-specific [*TP63* (ΔNp63 isoform), *EGFR*, and *STAT6*] histone acetylation and methylation status in alveolar epithelial cells (AECs) obtained from asthmatic (n = 5) and healthy (n = 5) non-transplantable teenage human lung donors	Higher global H3K18ac and H3K9me3 levels were observed in asthmatic subjects. Higher association of H3K18ac (but not H3K9me3) around the transcription start sites of *TP63* (ΔNp63 isoform), *EGFR*, and *STAT6* was found in asthmatics. Non-significant increase in protein expression of those three genes was detected in AECs treated with HDACi (TSA)
Cahill et al. [62]	Analysis of the effect H3K27ac at the *PTGER2* promoter on EP2-expression in polyp fibroblasts obtained from aspirin-exacerbated respiratory disease (AERD) patients (n = 18), aspirin-tolerant and asthma-free control subjects with chronic rhinosinusitis and polyposis (aspirin-tolerant controls; n = 9), and healthy control subjects undergoing sinus surgery for concha bullosa (n = 8)	Independent of disease state, the levels of H3K27ac at *PTGER2* were variable (in contrast to the H3K27ac at *PTGER4* that were constant across samples) and correlated significantly with EP2 receptor expression (*PTGER2* mRNA levels). After HDACi (TSA) treatment, *PTGER2* mRNA levels increased in fibroblasts obtained from subjects with AERD or aspirin-tolerant controls but not in those from healthy controls
Marwick et al. [69]	Analysis of oxidant-associated inflammation (such as observed in severe asthma)-induced H3S10ph at promoters of inflammatory genes on the anti-inflammatory effect of corticosteroids (CS)	The induction of H3S10ph at promoters of the *IL6*, *CCL2*, and *CXCL8* in alveolar macrophages from severe asthmatic patients was not reduced by CS. Application of a selective p38α MAPK inhibitor, SB239063, and IKK-2 inhibitor, TPCA-1, resulted in reduced induction of H3S10ph; this inhibitory effect was even stronger when SB239063 or TPCA-1 were combined with CS
Borriello et al. [68]	Analysis of the effect of IL-3 and IL-4 on STAT5 and STAT6 (respectively) binding and H3ac at the *CCL17* locus, encoding CCL17, a marker of the alternative activation of human monocytes	IL-3 and IL-4 together increased H3ac at *CCL17* locus. IL-4 alone but not in combination with IL-3 induced STAT6 binding at the *CCL17* locus. No identifiable STAT5 binding at the *CCL17* locus was observed
Harb et al. [72]	Comparison of H3ac and H4ac levels at Th1, Th2 and Treg-cell-related genes in isolated CD4+ T-cells obtained from children with allergic asthma (n = 14) and form healthy control children (n = 18)	Higher H3ac and H4ac levels at the *IL13* locus observed in children with allergic asthma when compared to healthy controls. This difference correlated with higher IL-13 protein levels in supernatants of anti-CD3/CD28-stimulated PBMCs of allergic asthmatic children compared with healthy controls. The levels of H3ac at the *FOXP3* locus were higher in allergic asthmatics than in healthy controls
Clifford et al. [54]	Comparison of ASMCs H3ac and H4ac, H3K9me2/3, H3K4me2/3, and DNA methylation levels at the *CXCL8* promoter between asthmatic (n = 7) and non-asthmatic subjects (n = 6)	No differences in H4ac, H3K9me2/3 and H3K4me2/3 or in DNA methylation levels were detected between asthmatic and non-asthmatic subjects. However, significantly higher H3ac levels, specifically H3K18ac, and higher binding of bromodomain-containing HATs, p300 and PCAF, were observed in asthmatics. BET inhibitors reduced CXCL8 secretion
Perry et al. [55]	Analysis of the effect of BET bromodomains on the TGF-β-induced proliferation and cytokine release in ASMCs [from healthy (n = 9), non-severe asthmatic (n = 9), and severe asthmatic (n = 9) subjects]	An inhibition of FCS + TGF-β-induced cell proliferation as well as IL-6 and CXCL8 expression (*IL6* and *CXCL8* mRNA levels, and IL-6 and CXCL8 protein release) after treatment with BET bromodomain mimics JQ1/SGCBD01 and I-BET762 was observed. A higher concentration of both mimics was needed depending on the asthma severity
Comer et al. [56]	Comparison of H3ac and H4ac levels at the *COX2* locus in ASMCs obtained from asthmatic (n = 7) and non-asthmatic subjects (n = 5) before and after treatment with cytomix (IL-1β, TNF-α, and IFN-γ)	No differences in histone acetylation between the asthmatic and non-asthmatic ASMCs were detected. Moreover, no differences in histone acetylation were observed after the cytomix treatment in both groups. Higher COX-2 protein levels were found in asthmatics, most likely due to posttranslational regulation (miR-155)
Seumois et al. [73]	Comparison of global H3K4me2-marked *cis*-regulatory regions in naïve, Th1, and Th2 CD4 + T-cells obtained from asthmatic (n = 12) and non-asthmatic subjects (n = 12)	Differential enrichment of H3K4me2 was observed in 200 enhancer regions in the three cell types when comparing asthmatic vs. non-asthmatic subjects. 163 of 200 asthma-associated enhancers were Th2-specific and 84 of them contained binding sites for transcription factors involved in T-cell differentiation (e.g. GATA3, TBX21 and RUNX3)
Zhang et al. [67]	Analysis of the anti-inflammatory and steroid-enhancing effects of vitamin D in monocytes obtained from patients with steroid-resistant (SR; n = 11) and with steroid-sensitive (SS; n = 8) asthma	Significant increase in H4ac levels at the glucocorticoid response element upstream of the *DUSP1* locus (encoding for MKP-1) occurred after treatment of the monocytes obtained from both SS and SR patients with dexamethasone. Preincubation with calcitriol resulted in a significant enhancement in the dexamethasone-mediated H4ac. Higher H4ac was observed in SS patients than in SR patients
Kobayashi et al. [85]	Analysis of the effect of passive smoking on HDAC2 expression and activity and on corticosteroid sensitivity in alveolar macrophages (AMs) obtained from children with severe asthma exposed (n = 9) or not exposed (n = 10) to passive smoking	Significantly lower HDAC2 protein expression and HDAC2 activity in passive smoking-exposed group. Higher levels of phosphorylation of Akt1 in AMs of the passive smoking-exposed group correlated negatively with HDAC2 activity. Significant inhibition (40%) of the TNF-α-induced CXCL8 production in AMs from subjects not exposed to passive smoking after treatment with dexamethasone was observed. In contrast, no significant inhibition was detected in AMs from passive smoking-exposed subjects
Cho et al. [63]	Analysis of the effect of HDACi (TSA) on myofibroblast differentiation and extracellular matrix (ECM) accumulation in nasal polyposis (18 patients)	Suppression of the TGF-β1-induced myofibroblast differentiation and ECM production after treatment with TSA was observed (α-SMA, fibronectin, and collagen type I expression at both RNA transcript and protein levels were diminished). This was due to decreased expression of both HDAC2 and HDAC4 and increased histone four acetylation
Clifford et al. [57]	Comparison of H3ac and H4ac, H3K4me3, H3K9me3, and DNA methylation levels at the *VEGFA* promoter in ASMCs obtained from asthmatic (n = 7) and non-asthmatic (n = 6) subjects	No differences in the DNA methylation or H3ac or H4ac at the *VEGFA* locus were identified between asthmatic and non-asthmatic subjects. Nevertheless, slightly but consistently higher H3K4me3 (a mark associated with transcription activation) levels and much lower H3K9me3 (a mark associated with transcription repression) levels were detected in ASMCs obtained from asthmatic subjects. The low H3K9me3 was an aftermath of abortive recruitment of G9a in asthmatic ASMCs. After treatment with BIX-01294, a histone methyltransferase (HMT) G9a inhibitor, VEGF expression increased in cells obtained from non-asthmatics to near asthmatic levels
Cho et al. [64]	Analysis of the effect of HDACi (TSA) on myofibroblast differentiation and ECM accumulation in nasal polyposis (7 patients with chronic rhinosinusitis with nasal polyps vs. normal inferior turbinate tissues)	Increased expression (mRNA/protein) of HDAC2, α-SMA and TGF-β1 was observed in nasal polyp tissues. In addition to its suppressive effect on the TGF-β1-induced myofibroblast differentiation and ECM production (α-SMA and collagen type I expression was diminished after TSA treatment), TSA also reversed TGF-β1-induced morphological changes in nasal polyp-derived fibroblasts (NPDFs). Inhibition of the HDAC2 expression and increased H3ac and H4ac were also a TSA application aftermath. Inhibiting HDAC2 with siRNA had a similar effect to TSA. This TSA suppressive effect was produced by inhibiting the TGF-β1-induced phosphorylation and translocation of Smad2/3 (to nucleus). Moreover, TSA blocked NPDFs proliferation with no cytotoxic effects
Kuo et al. [101]	Analysis of the effect of iloprost, a prostaglandin I2 (PGI_2_) analog, on the expression of TNF-α in human myeloid dendritic cells (mDCs) obtained from 6 healthy subjects via histone modifications	Downregulation of the poly I:C-induced H3K4me3 in the *TNF* promoter region was observed after treatment with iloprost. This suppressive effect was produced by inhibiting the poly I:C-induced translocation of H3K4-specific methyltransferase MLL (mixed lineage leukemia) and WDR5 (WD repeat domain 5) proteins from the cytoplasm to the nucleus
Yang et al. [65]	Analysis of the effect of TGF-β on the ADAM33 mRNA expression and ADAM33 protein in bronchial fibroblasts obtained from asthmatic (n = 7) and healthy (n = 6) subjects during their differentiation into myofibroblasts via histone modifications	Although no difference in transcript levels of *ADAM33* between asthmatic and healthy control cells was detected, a suppression of *ADAM33* mRNA expression after treatment with TGF-β was observed. This was caused by chromatin condensation at the *ADAM33* promoter via histone modification (deacetylation of H3ac, demethylation of H3K4, and hypermethylation of H3K9) and not by DNA methylation. Stimulation of ADAM33 ectodomain shedding was also caused by TGF-β

**Fig. 1:**
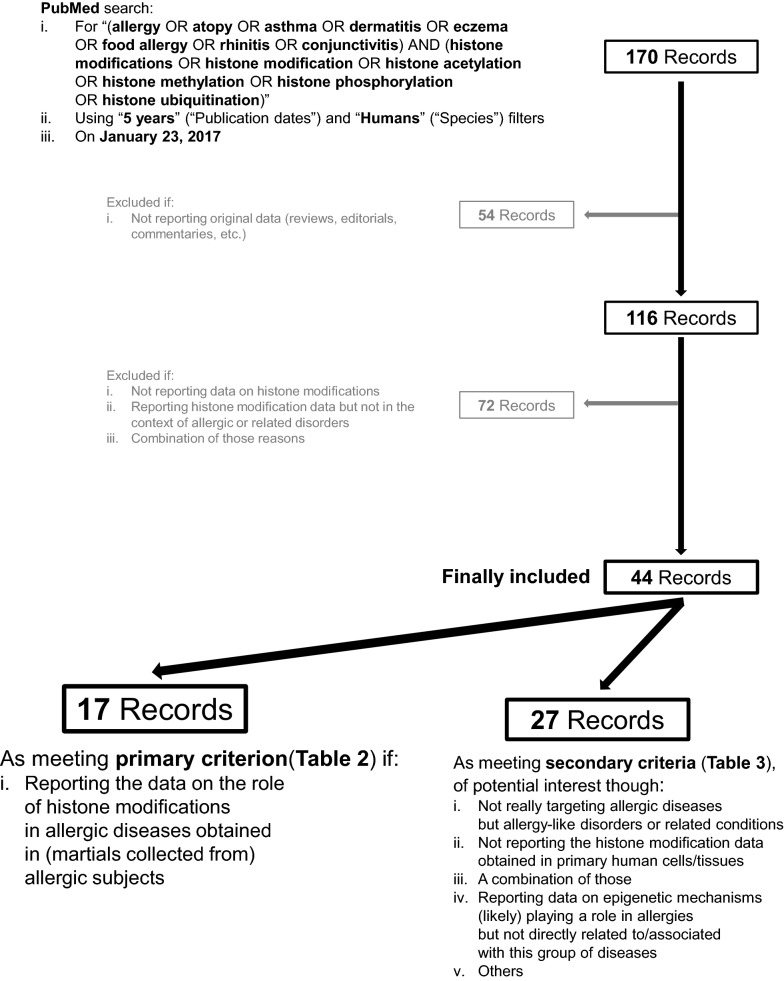
Strategy of systematic literature search and its results

For criteria of the selection, please, refer to Fig. 1 and “Systematic search: methodology” section of “Main text”

α-SMA, denotes alpha smooth muscle actin; *ADAM33*, ADAM metallopeptidase domain 33 (ADAM33) gene; ASMC, airway smooth muscle cell; Akt1, RAC-alpha serine/threonine-protein kinase; BET, bromodomain and extra-terminal (proteins); *CCL2/17*, C–C motif chemokine ligand 2 (CCL2/17) gene; CB, cord blood; *COX2*, cytochrome c oxidase subunit II (COX2) gene; *CXCL8/10*, C–X–C motif chemokine ligand 8/10 (CXCL8/10) gene; *DUSP1*, dual specificity phosphatase 1 gene; *EGFR*, epidermal growth factor receptor (EGFR) gene; FCS, fetal calf serum; *FOXP3*, forkhead box P3 (FOXP3) gene; G9a, euchromatic histone-lysine *N*-methyltransferase 2; *GATA3*, GATA binding protein 3 (GATA3) gene; HAT, histone acetyltransferase; HDACi, histone deacetylase (HDAC) inhibitor; *IFNG*, interferon gamma (IFN-γ) gene; IKK-2, IĸB kinase 2; IL-1β, interleukin 1 beta; *IL6/9/13*, interleukin 6 (IL-6/-9/-13) gene; MAPK, p38 mitogen-activated protein kinase; MKP-1, MAPK phosphatase 1; p300, E1A binding protein p300; PBMC, peripheral blood mononuclear cell; PCAF, P300/CBP-associated factor; *PRKCZ*, protein kinase C zeta (PKCζ) gene; *PTGER2/4*, prostaglandin E receptor 2/4 (EP2/4) gene; RUNX3, runt-related transcription factor 3; STAT1/3/5/6, signal transducer and activator of transcription 1/3/5/6; *TBX21*, T-box 21 (TBX21) gene; TGF-β, transforming growth factor beta; Th1/2/17, cells, helper T-cells/T-helper cells type 1/2/17; *TNF*, tumor necrosis factor (TNF-α) gene; *TP63*, tumor protein p63 (TP63) gene; Treg, cells, regulatory T-cells; TSA, trichostatin A; *VEGFA*, vascular endothelial growth factor A (VEGF) gene

For the remaining abbreviations please refer to Table 1

**Table 3:** Studies on the role of histone modifications in allergic diseases meeting the secondary selection criteria

Study	Major epigenetic findings (in the context of major study results)
Zhong et al. [102]	Significantly decreased expression of Th2-related cytokines (IL-4, IL-5) in human CD4+ T-cells and PBMCs was observed after transfection with chemically synthesized PIWI interacting RNA (piRNA), piR30840. Accordingly, antisense inhibition of the endogenous piR30840 resulted in CD4+ T-cells with upregulated IL-4 expression. As no differences in histone methylation levels at the *IL4* promoter region between piR30840-transfected and -untransfected CD4+ T-cells were detected, the functioning of piR30840 was not attributed to this type of histone modification but rather to pre-mRNA decay through nuclear exosomes
Zheng et al. [103]	After treatment with TSA, a significant increase of the (lower) transcription of STAT3-dependent genes (*IL17A* and *IL22*) in IL-23-stimulated PBMCs from chronic mucocutaneous candidiasis (CMC) patients (n = 16) (back) to control (n = 37) levels was observed. Conversely, TSA significantly decreased the enhanced transcription of STAT1-dependent genes (*CXCL10* and *IRF1*) in IFN-α-stimulated gain-of-function STAT1-mutation Epstein-Barr virus (EBV)-transformed B cell line back to control levels
Vicente et al. [104]	Noticeable regulatory activity characterized by H3K4me1/2 peak identified four putative regulatory elements (PREs) in the 8q21 core region of genetic association (with allergies) in lymphoblastoid cells. Significant correlation between the allergy-associated SNP rs7009110 located on the 8q21 and *PAG1* expression
Naranbhai et al. [105]	Significant enrichment of peak cis-eQTL was detected in DNA-hypomethylated regions, and in regions marked by H3K4me3 (associated with promoter activity), H3K27ac and H3K4me1 (associated with active or poised enhancers), and H3K36me3 (associated with gene activation) in neutrophils. Correspondingly, a depletion of peak cis-eQTL was observed in DNA-hypermethylated regions and in regions marked by H3K27me3 and H3K9me3 (repressive histone modifications). A SNP rs2240335, an eQTL in *PADI4* (a locus associated with rheumatoid arthritis), was shown to be located within regions marked by H3K27ac and H3K4me1
Lin et al. [106]	Increased H4ac levels at the *VCAM1* promoter region correlating with higher *VCAM1* expression (protein/mRNA) and promoter activity in human tracheal smooth muscle cells were observed upon pretreatment with ET-1, mediated via Elk-1/p300 interaction, in an ET receptor/Src/RTK/PI3K/AKT/p42/p44 MAPK-dependent manner
Castellucci et al. [70]	Ablation of the IL-10-mediated deacetylation of H4ac at the *CXCL8* promoter in LPS-stimulated monocytes was observed after CI-994, a class I HDACi, treatment. Concordantly, IL-10-mediated inhibition of *CXCL8* transcription in LPS-stimulated PBMCs obtained from COPD patients (n = 6), due to reduced HDAC2 expression, was dampened compared to acute respiratory failure patients (n = 4) and healthy controls (n = 6)
Hsieh et al. [71]	Noticeable suppression of the LPS-induced MDC/CCL22 expression in THP-1 cells (human monocyte cell line) and human primary monocytes was observed after treatment with sesamin, a class of phytoestrogen isolated from sesame seed *Sesamum indicum*, due to dampened recruitment of a HAT, CREBBP, and a subsequent decrease in H3ac and H4ac levels at the *CCL22* promoter
Sharma et al. [107]	Significant enrichment of active histone marks H3K4me1 and H3K27ac at the regions near to two asthma-associated SNPs in the *FADS2* (rs968567) and the *NAGA* (rs1801311) loci was observed in three different cell lines: Jurkat (human T lymphocyte cell line), Beas-2B (human bronchial epithelial cell line) and A549 (human lung epithelial cell line). Consistently, both SNPs showed significant enrichment of formaldehyde-assisted isolation of regulatory elements (FAIRE) signals in all three cell lines
Escobar et al. [108]	Markedly increased Jumonji, AT Rich Interactive Domain 2 (Jarid2), a DNA-binding protein that recruits the polycomb repressive complex 2 (PRC2) to chromatin, and H3K27me3 islands were detected throughout the genome (among other regions, within 10 kb of the *Il22*, *Il10*, and *Il9* loci) in miR-155-deficient compared to wild-type mouse Th17 cells
Huber et al. [74]	Significantly elevated levels of the repressive H3K27me3 mark at the conserved noncoding sequence (CNS-1) located 5 kb upstream of exon 1A of the *GATA3* gene in primary naïve human CD4+ T-cells after treatment with IFN-α (both, with or without IL-4 co-stimulation) were observed, resulting in a consequential inhibition of GATA3 binding to exon 1A (i.e. down-regulation of a positive-feedback loop of *GATA3/*GATA3 autoactivation and thus reduced gene expression)
Gschwandtner et al. [109]	When compared to both neonatal and adult human keratinocytes (KCs), fetal human KCs produced more AMPs, and had lower global H3K27me3 levels and higher expression of a histone demethylase *JMJD3*. *JMJD3* knockdown with siRNA led to an increase in H3K27me3 levels and lower AMP production
Coward et al. [58]	Markedly increased H3K9me3 and H3K27me3 levels at the *COX2* promoter were detected in primary human fibroblasts from IPF lung (stimulated or not with the COX2 inducer, IL-2β) when compared to fibroblasts from non-fibrotic lung, which were related to the recruitment of HMTs, G9a and EZH2. Substantially decreased H3K9me3 and H3K27me3, and increased H3ac and H4ac levels at the *COX2* promoter resulting in *COX2* mRNA and protein re-expression were observed in fibroblasts from IPF lung after treatment with G9a or EZH2 inhibitors (more significant effect with IL-2β stimulation)
Sanders et al. [59]	Markedly increased H3K9ac and decreased H3K9me3 at the pro-apoptotic *BAK1* gene accompanied with increased expression (mRNA) and substantially decreased H3K9ac and increased H3K9me3 levels at the anti-apoptotic *BCL2L1* gene accompanied by a decrease in gene expression (mRNA) were observed in primary human IPF fibroblasts after treatment with a HDACi, SAHA
Han et al. [110]	Significantly reduced H3K27me3 levels at the *ALOX15* promoter were detected in A549 cells after treatment with IL-4, coinciding with higher *ALOX15* mRNA levels. More potent induction of *ALOX15* was observed in human monocytes. Significant increase in H3K27me3 levels after depletion of a lysine demethylase UTX (with siRNA) was observed, resulting in reduced IL-4-induced *ALOX15* expression. Inhibition of the IL-4-mediated *ALOX15* expression in UTX-depleted human monocytes with no changes in the H3K27me3 levels when compared to control monocytes was found
Lakshmi et al. [111]	Markedly reduced expression of PPARy correlated with downregulated HDAC2 expression in lung and human bronchial epithelial cells (HBE) obtained from COPD patients compared with healthy controls. Noticeable suppressed GR-α expression in H292 cells (human lung epithelial cell line) after CSE treatment due to dampened expression of PPARy and HDAC2 was also observed. The suppressive effect of CSE on GR-α and HDAC expression was attenuated with PPARy agonists
Zhang et al. [60]	Downregulation of *COL3A1* expression in primary human IPF myofibroblasts with concomitant increases of H3ac, H4ac, H3K9ac, and H3K27me3 levels at *COL3A1* promoter was observed after SAHA treatment
Wiegman et al. [112]	Significantly increased HAT activity levels and decreased HDAC2 activity levels (due to protein modification: nuclear phosphorylation and cytoplasmic carbonylation) were observed in lung extracts obtained from mice after ozone exposure compared to air-exposed animals
Che et al. [113]	Inhibition of the sphingosine-1-phosphate (S1P)-induced IL-6 secretion by primary human ASMCs via MKP-1-mediated repression of MAPK-driven activation of mitogen and stress-activated protein kinase 1 (MSK1) and phosphorylation at H3S10 (H3S10ph; global; putatively at *IL6* promoter as well) was observed after the treatment of the cells with dexamethasone
Liu et al. [61]	Significantly higher H3K9ac levels at the *TERT* promoter region were detected in primary human lung fibroblasts from patients with IPF (n = 70) compared with healthy control subjects (n = 117). After treatment of the cells with a HDACi (TSA), significantly elevated *TERT* mRNA and TERT protein levels (time and dose-dependent increase) were detected in both human lung fibroblasts from patients with IPF and mouse lung fibroblasts from a murine model of bleomycin-induced pulmonary fibrosis
Gerasimova et al. [75]	Noticeable enrichment of asthma-associated non-coding SNPs and H3K4me1 peaks (enhancers) in the Th2 cytokine locus of CD4+ T-cells (when compared with other cell/tissue types) was observed
Li et al. [76]	Significantly decreased LAT expression consistent with decreased H3ac and H4ac levels and increased H3K9me2 levels at the LAT (*Lat*) upstream region in lung T-cells obtained from ovalbumin-induced allergic airway inflammation rat model when compared with control animals were found. H3ac and H4ac as well as LAT expression noticeably increased after treatment of the cells with TSA
Luo et al. [77]	Increased global H3ac and H3K4me levels were observed in PBMCs of HSP patients with kidney damage (n = 16) compared to HSP patients without kidney damage (n = 8) and healthy controls (n = 22). Higher CD4 + T-cell H3ac and H3K4me3 levels were detected at *IL4* regulatory regions of HSP patients when compared to control subjects. Higher expression (mRNA) of several HATs (CREBBP, PCAF, and p300) and HMTs (SETD1A, SETDB1, SUV39H1, and SUV39H2) and lower expression of a few HDACs (HDAC1, HDAC2, HDAC3, and SIRT1) were found in PBMCs of both HSP groups compared to healthy controls
Kallsen et al. [114]	Markedly increased H3ac and H3K4me3 levels at the *DEFB1* promoter in A549 cells coinciding with enhanced DEFB1 expression after treatment with class I HDACi (MS-275) were found
Han et al. [78]	Noticeable DNA hypomethylation at 26 unique regions (10–70 kb) was found in naïve CD4+ T-cells obtained from psoriasis patients (n = 12) compared to atopic dermatitis patients (n = 15) and healthy controls (n = 10). These regions coincided incidentally with various strong epigenomic signals, including histone modifications (H3K4me1, H3K27ac, and H3K4me3), and transcription factor binding sites
Robertson et al. [115]	Significantly increased HDAC activity (only at the protein level) was observed in ARNT-depleted *N*-TERT keratinocytes (15–20% increase) and in ARNT-depleted immortalized human HaCaT keratinocytes (~ 50% increase). After treating ARNT-depleted *N*-TERT keratinocytes with TSA, the negative effect of ARNT-deficiency on transcription of amphiregulin gene (*AREG*), an important ligand of EGFR, was abolished
Vazquez et al. [116]	A novel inducible hypersensitive region was identified in human and mouse lymphocytes that is located in intron I of *CD69*. H3K4me2, H3ac, H3K14ac, and/or H3K4ac levels within this region were found to be dynamically regulated during thymocyte development and/or to be constitutively high in mature T lymphocytes
Zijlstra et al. [117]	Reduced glucocorticoid sensitivity and dampened HDAC activity were observed in 16HBE cells (human bronchial epithelial cell line) after treatment with IL-17A. Overexpression of HDAC2 reversed IL-17A-induced glucocorticoid insensitivity

For criteria of the selection, please, refer to Fig. 1 and “Systematic search: methodology” section of “Main text”

*ALOX15*, arachidonate 15-lipoxygenase (ALOX15) gene; AMP, antimicrobial peptide; ARNT, aryl hydrocarbon receptor nuclear translocator; *BAK1*, BCL2 antagonist/killer (Bak) gene; *Bcl*-*xL*, B-cell lymphoma-extra large (Bcl-xl) gene; CBP, CREB binding protein; *COL3A1*, collagen type III alpha 1 chain (COL3A1) gene; COPD, chronic obstructive pulmonary disease; CREBBP, CREB binding protein; CSE, cigarette smoke extract; *DEFB1*, defensin beta 1 (DEFB1) gene; eQTL, expression quantitative trait loci; Elk-1, ETS transcription factor; ET-1, endothelin-1; EZH2, enhancer of zeste 2 polycomb repressive complex 2 subunit; *FADS2*, fatty acid desaturase 2 (FAD2) gene; GR-α, glucocorticoid receptor alpha; HMT, histone methyltransferase; HSP, Henoch-Schönlein purpura; *IL17A*/22, interleukin 17A/22 (IL-17A/-22) gene; *Il9/10/22*; mouse interleukin 9/10/20 (il-9/-10/-22) gene; IL-23/2β, interleukin 23/2β; INF-α, interferon alpha; IPF, Idiopathic pulmonary fibrosis; IRF1, interferon regulatory factor 1; LAT, linker for activation of T-cells; *MDC*/*CCL22*, macrophage-derived chemokine/C–C motif chemokine ligand 22 (*CCL22*) gene; *NAGA*, alpha-*N*-acetylgalactosaminidase (NAGA) gene; *PADI4*, peptidyl arginine deiminase 4 (PADI4) gene; *PAG1*, phosphoprotein membrane anchor with glycosphingolipid microdomains 1 (PAG1) gene; PCAF, lysine acetyltransferase 2B; PI3K, phosphatidylinositol-4,5-bisphosphate 3-kinase; PPARy, peroxisome proliferator activated receptor gamma; RTK, Receptor tyrosine kinase; SAHA, suberoylanilide hydroxamic acid; siRNA, small interfering RNA; SNP, single-nucleotide polymorphism; Src, SRC proto-oncogene, non-receptor tyrosine kinase; *TERT*, telomerase transcriptase (TERT) gene; *VCAM1*, vascular cell adhesion molecule-1 (VCAM-1) gene

For the remaining abbreviations please refer to Tables 1 and 2

#### Histone acetylation

Histone acetylation status is regulated by two groups of enzymes exerting opposite effects, histone acetyltransferases (HATs) and histone deacetylases (HDACs). HATs catalyze the transfer of an acetyl group from acetyl-CoA to an amino acid group of the target lysine residues in the histone tails, which leads to the removal of a positive charge on the histones, weakening the interaction between histones and (negatively charged phosphate groups of) DNA. This in turn typically makes the chromatin less compact and thus more accessible to the transcriptional machinery. HDACs remove acetyl groups from histone tail lysine residues and thereby work as repressors of gene expression [5, 14, 21–24].

HATs are classified into five (or sometimes six) families. The GCN5-related *N*-acetyltransferase (GNAT) family comprises KAT2A and KAT2B enzymes. They are involved in acetylation of histones and transcription factors and thus cell cycle regulation, and DNA replication and repair [25, 26]. Moreover, these enzymes have been recently identified to be important for centrosome function as well [27]. The MYST family is in turn composed of KAT6A/MOZ/MYST3, KAT6B/MORF/MYST4, KAT7/HBO1/MYST2, KAT8/hMOF/MYST1, and KAT5/Tip60. It contributes to transcription regulation and is also responsible for DNA repair [28–30]. Interestingly, autoacetylation of MYST family protein enzymes participates in their regulation, which makes them distinct from other acetyltransferases, drawing at the same time similarities to the phosphoregulation of protein kinases [31, 32]. The other HAT families are much smaller. KAT3A and KAT3B enzymes belong to p300/CBP family, and KAT4/TAF1/TBP and KAT12/TIFIIIC90 are members of the general transcriptional factor-related HAT family [23, 28, 33]. Steroid receptor co-activators family comprises KAT13A/SRC1, KAT13B/SCR3/AIB1/ACTR, KAT13C/p600, and KAT13D/CLOCK [23, 34]. Finally, KAT1/HAT1 and HAT4/NAA60 are cytoplasmic HATs [23].

Eighteen enzymes belonging to the HDAC superfamily have been identified. They are further subdivided into four classes, including class I (HDAC1, HDAC2, HDAC3, and HDAC8), class IIa (HDAC4, HDAC5, HDAC7, and HDAC9), class IIb (HDAC6 and HDAC10), class III, so-called sirtuins (SIRTs; SIRT 1–7; enzymes evolutionally and mechanistically different from the other HDACs), and class IV (HDAC11) [35–37]. Class I HDACs are characterized by a ubiquitous nuclear expression in all tissues, class IIb HDACs are present both in the nucleus and cytoplasm, and class IIa HDACs show mainly cytosolic localization. Not much is known about HDAC11, and sirtuins which localize in nucleus, cytosol and/or mitochondria [36].

#### Histone methylation

Histone methylation is mediated by histone methyltransferases (HMTs), including lysine methyltransferases (KMTs) and arginine methyltransferases (PRMTs), and histone demethylation by histone demethylases (HDMs).

Whereas acetylation of the histone lysine affects the electrical charge of the histones and thus their interaction with DNA, methylation of histone lysine or arginine does not affect this electrostatic bond, but instead indirectly influences the recruitment and binding of different regulatory proteins to chromatin [19, 38, 39]. HMTs can transfer up to three methyl groups from the cofactor S-adenosyl-l-methionine (SAM) to lysine or arginine residues of the histones [19, 38]. More than 50 human KMTs are known at the moment, which, based on their catalytic domain sequence, can be further subdivided into the SET domain-containing and the DOT1-like protein family, the latter having only one representative in humans, with a catalytic domain structurally more similar to the PRMTs [19, 38, 39]. KMTs are more specific than HATs and they generally target a specific lysine residue. Methylation of H3K4 residue (for the description of histone modifications including their location, character and effect on transcription, please, refer to Table 1) is mediated in mammals by KMTs such as KMT2A/MLL1, KMT2A/MLL2, KMT2F/hSET1A, KMT2G/hSET1B, or KMT2H/ASH1. Examples of KMTs responsible for H3K9 methylation include KMT1A/SUV39H1, KMT1B/SUV39H2, KMT1C/G9a, or KMT1D/EuHMTase/GLP. H3K36 methylation is catalyzed by e.g. KMT3B/NSD1, KMT3C/SMYD2, or KMT3A/SET(D)2. KMT6A/EZH2 methylates H3K27, andKMT4/DOT1L targets H3K79. Etc. [19, 38, 39].

Based on the catalytic mechanism and sequence homology, HDMs can be divided into two classes. Firstly, amine-oxidase type lysine-specific demethylases (LSDs or KDM1 s), including KDM1A/LSD1/AOF2 and KDM1B/LSD2/AOF1. These remove the methyl groups from mono- and dimethylated H3K4. Secondly, the JumonjiC (JMJC) domain-containing HDMs, in turn, catalyze the demethylation of mono-, di-, and trimethylatedlysine residues at various histone amino acid residues. Over thirty members of this group can be further subdivided, based on the JMJC domain homology, into seven/eight subfamilies (KDM2–7/8) [19, 38–41].

#### Histone phosphorylation

Histone phosphorylation status is controlled by two types of enzymes having opposing modes of action. While kinases add phosphate groups, phosphatases remove the phosphates [13, 15]. At least three functions of phosphorylated histones are known, DNA damage repair, the control of chromatin compaction associated with mitosis and meiosis, and the regulation of transcriptional activity (similar to histone acetylation) [13, 15]. In comparison to histone acetylation and methylation, histone phosphorylation works in conjunction with other histone modifications, establishing the platform for mutual interactions between them. This cross-talk results in a complex downstream regulation of chromatic status and its consequences [13, 15, 42]. For example, histone H3 phosphorylation (specifically H3S10ph) can directly affect acetylation levels at two amino acid residues of the same histone (H3K9ac and H3K14ac) [43, 44]. Furthermore, H3S10ph can induce transcriptional activation by interaction with H4K16ac [42].

#### Histone ubiquitination

Protein ubiquitination is an important post-translational modification that regulates almost every aspect of cellular function in many cell signaling pathways in eukaryotes. Ubiquitin is an 8.5 kD protein which is conjugated to substrate proteins by the ubiquitin–proteasome system thereby regulating the stability and turnover of the target proteins. Histone ubiquitination is carried out by histone ubiquitin ligases and can be removed by ubiquitin-specific peptidases, the latter known as deubiquitinating enzymes (DUBs) [45–47]. Monoubiquitination has a critical role in protein translocation, DNA damage signaling, and transcriptional regulation. Histone 2A monoubiquitination (H2Aub) is more often associated with gene silencing. Monoubiquitination of histone 2B (H2Bub) is typically correlated with transcription activation. Polyubiquitination marks the protein for degradation or activation in certain signaling pathways [45–48]. Similarly to histone phosphorylation, there is also cross-talk between histone ubiquitination and other histone modifications [46–48]. For instance, monoubiquitination of histone H3 is able to induce acetylation of the same histone [49].

#### Epigenetic readers

In addition to epigenetic writers, i.e. enzymes adding epigenetic marks on histones (HATs, HMTs/KMTs, PRMTs, kinases, ubiquitin ligases) and epigenetic erasers (HDACs, HDMs/KDMs, phosphatases, DUBs), there are also epigenetic readers, which are the molecules that recognize and bind to the epigenetic marks created by writers, thereby determining their functional consequences. They include proteins containing bromodomains, chromodomains, or Tudor domains [50, 51]. Some enzymes with primary activities different from epigenetic reading possess bromodomains as well, for example certain HATs [51].

### Systematic search: methodology

In order to cover the area of interest, a systematic literature search was conducted (Fig. 1). In brief, On January 23, 2017, the PubMed database (http://www.ncbi.nlm.nih.gov/pubmed) was searched by using the input “(allergy OR atopy OR asthma OR dermatitis OR eczema OR food allergy OR rhinitis OR conjunctivitis) AND (histone modifications OR histone modification OR histone acetylation OR histone methylation OR histone phosphorylation OR histone ubiquitination)”, restricting the results with “5 years” (“Publication dates”) and “Humans” (“Species”) filters, which yielded a total of 170 hits. These were subsequently subjected to full text-based screening to exclude articles not reporting original data (reviews, editorials, commentaries, etc.), which resulted in elimination of 54 publications. From the remaining 116 papers, a further 72 were excluded as not being directly or at least indirectly relevant to the topic of the present review (not reporting data on histone modifications, reporting histone modification data but not in the context of allergic or related disorders, or both). The remaining 44 articles were divided into two groups. The group that met the primary selection criterion contained 17 papers reporting the data on the role of histone modifications in allergic diseases obtained in material collected from allergic subjects and thus directly relevant to allergies is presented in Table 2. Another 27 articles of potential interest comprised the additional group (Table 3). These did not necessarily target allergic disorders but allergy-like diseases or related conditions, did not report histone modification data obtained in primary human cells/tissues, or indeed a combination of those. This included also those reporting data on epigenetic mechanisms likely playing a role in allergies but not directly related to/associated with this group of diseases.

### Systematic search: review

Epigenetic mechanisms are thought to play an important regulatory role in allergic inflammation and the development of allergic disorders. DNA methylation is the classical epigenetic modification that has been most widely studied in this context. However, histone modifications, which contribute to the lineage commitment, differentiation and maturation of immune cells, including those strongly involved in allergic inflammation such as CD4+ T-helper (Th) cells, are likely to play a crucial role in the predisposition to developing atopic diseases as well as in the effector phase of allergic inflammation [5, 6, 10, 52, 53]. Indeed, our systematic search identified a number of recent studies that sought to define the relationships between histone modifications and allergic inflammation or related immune mechanisms, and/or allergic diseases or disorders sharing some of the pathophysiology. The results reported in those 44 original articles are summarized in Tables 2 and 3.

Several studies investigated the relationships between histone modifications in airway smooth muscle cells (ASMCs) and the respiratory tract allergic inflammatory disease. For instance, increased binding of bromodomain-containing HATs [E1A binding protein p300 (p300) and p300/CBP-associated factor (PCAF)] accompanied by significantly higher H3ac levels (specifically H3K18ac) at the C–X–C motif chemokine ligand 8 (CXCL8) gene (*CXCL8*) promoter were observed in ASMCs obtained from asthmatics compared to healthy controls [54]. Furthermore, treatment of cultured cells with bromodomain and extra-terminal (BET) protein inhibitors reduced CXCL8 secretion [54]. The application of BET bromodomain mimics reduced in turn fetal calf serum plus transforming growth factor beta (TGF-β) -induced ASMC proliferation and interleukin 6 (IL-6) gene (*IL6*) and *CXCL8* expression, with the required dose depending on asthma severity of cell donor [55]. On the other hand, no differences in H3ac and H4ac levels at the cytochrome c oxidase subunit II (COX2) gene (*COX2*) between the asthmatic and non-asthmatic ASMCs were detected, regardless of whether they were stimulated with proinflammatory cytokines [56]. Although asthmatic and non-asthmatic ASMCs did not differ in their H3ac or H4ac levels at the vascular endothelial growth factor A (VEGF) locus (*VEGFA*), the cells obtained from affected individuals displayed slightly but consistently higher H3K4me3 and a low H3K9me3 levels [57]. Moreover, treatment with an inhibitor of a HMT (HMTi), euchromatic histone-lysine *N*-methyltransferase 2 (G9a), increased VEGF expression in non-asthmatic ASMCs to near asthmatic levels [57].

Histone modifications at several of the above-mentioned loci contribute also to the pathophysiology of some other inflammatory disorders of the lung. For example, H3K9me3 and H3K27me3 levels at the *COX2* promoter were found to be substantially higher in primary human fibroblasts isolated from lung tissue of idiopathic pulmonary fibrosis (IPF) patients compared to non-IPF fibroblasts. This was accompanied by the recruitment of HMTs, G9a and enhancer of zeste 2 polycomb repressive complex 2 subunit (EZH2) [58]. Interestingly, after treatment with G9a or EZH2 inhibitors, the levels of H3K9me3 and H3K27me3 markedly decreased and H3ac and H4ac levels increased at the *COX2* promoter [58]. Several other studies observed the involvement of histone modifications in the regulation of gene expression in (human) IPF lung (myo-) fibroblasts, the effects of which were sensitive to HDAC inhibitor (HDACi) treatment [59–61]. Histone acetylation and/or methylation in (myo-) fibroblasts were also demonstrated to regulate expression of the loci involved in the pathogenesis of nasal chronic rhinosinusitis and polyposis, such as prostaglandin E receptor 2 (EP2) gene (*PTGER2*) [62]. Furthermore, HDACi treatment influenced HDAC expression and histone acetylation at several loci, thus affecting nasal polyp myofibroblast differentiation and extracellular matrix production [63, 64]. Finally, although no differences in ADAM metallopeptidase domain 33 (ADAM33) gene (*ADAM33*) expression were observed between asthmatic and healthy control bronchial fibroblasts, treatment with TGF-β suppressed *ADAM33* mRNA expression through chromatin condensation related to deacetylation of H3ac, demethylation of H3K4, and hypermethylation of H3K9 at the *ADAM33* promoter [65]. Asthmatic and non-asthmatic histone acetylation levels were compared also in alveolar epithelial cells [66]. Global H3K18ac and H3K9me3 levels were higher in cells from asthmatics, which was also the case for gene-specific H3K18ac (but not H3K9me3) around transcription start sites of the loci encoding tumor protein p63 (*TP63*; ∆Np63 isoform), epidermal growth factor receptor (*EGFR*), and signal transducer and activator of transcription 6 (*STAT6*) [66]. The latter effect was ablated upon HDACi treatment [66].

Several studies were conducted on the biology of monocytes, the mechanisms of epigenetic modulation controlling production of cytokines, and their role in the onset/severity of allergic diseases. H4ac levels at the glucocorticoid response element upstream of the dual specificity phosphatase 1 gene (*DUSP1*) encoding for MAPK phosphatase 1 (MKP-1) substantially increased in dexamethasone-treated cells obtained from both steroid-sensitive and steroid-resistant asthmatics patients [67]. Furthermore, preincubation with calcitriol led to a significant enhancement of the dexamethasone-induced H4ac, with higher H4ac levels observed in monocytes obtained from steroid-sensitive than those from steroid-resistant individuals [67]. The involvement of histone acetylation or phosphorylation in regulation of gene expression in monocytes/macrophages was also demonstrated for C–C motif chemokine ligand 2/17/22 (*CCL2*/*17*/*22*)*, CXCL8*, or *IL6* loci [68–71]. In addition, in monocytes, histone modification changes were susceptible to pharmacological modification ex vivo, demonstrated by the effect of HDACi on *CXCL8* H4ac levels [70].

Several studies have focused on T-cells. For example, differences in H3ac and H4ac levels at the interleukin 13 (IL-13) gene (*IL13*) that were observed in CD4+ T-cells from children with allergic asthma and healthy controls correlated with serum IL-13 concentrations [72]. Differential enrichment of H3K4me2 in 200 *cis*-regulatory/enhancer regions in naïve, Th1, and Th2 CD4+ T-cells was observed between asthmatic and non-asthmatic subjects. Moreover, 163 of those 200 asthma-associated enhancers were Th2-specific and 84 of them contained binding sites for transcription factors involved in T cell differentiation [e.g. GATA binding protein 3 (GATA3), T-box 21 (TBX21) and RUNX3] [73]. Most of the other studies identified by our literature search were also supportive for the importance of histone modifications, such as acetylation and methylation, in (CD4+) T-cell biology and/or related pathophysiology of allergic disorders [74–78].

Some prenatal dietary exposures, previously demonstrated to modulate the infant’s immune responses and/or risk of allergy development in offspring [79–82], have recently been shown to be associated with the alterations in histone acetylation profiles in neonatal cells. For instance, cord blood (CB) CD4+ T-cells obtained from children born from mothers with highest serum folate levels during pregnancy were characterized by significantly higher histone H3ac and H4ac levels at the GATA3 gene (*GATA3*) promoter, markedly lower H4ac levels at the analogous region of the interferon gamma (IFNγ) gene (*IFNG*), and significantly higher interleukin-9 (IL-9) gene (*IL9*) promoter H4ac levels when compared to the lowest folate level group [83]. In CB CD4+ T-cells obtained from newborns of mothers supplemented with fish oil (ω − 3 fatty acids) during pregnancy in turn, significantly higher H3ac levels were observed at the protein kinase C zeta (PKCζ) gene (*PRKCZ*) and *IFNG* locus, and lower H3/H4ac levels at the IL-13 and TBX21 genes (*IL13* and *TBX21*, respectively) [84]. The infants from the fish oil-supplemented women were found to have lower risk of developing allergic diseases [81, 82].

Both passive (prenatal and postnatal) and active tobacco smoke exposures are a well-known extrinsic factors affecting the risk of allergic disorders, especially asthma, and this effect was found to be associated with (and thus is thought to be at least partly mediated by) changes in DNA methylation patterns [5, 6]. Exposure to passive smoking diminished corticosteroid sensitivity of alveolar macrophages obtained from children with severe asthma and was accompanied by lower HDAC2 expression and activity. This possibly explains the unfavorable effect [85] and suggests that histone modifications, specifically histone acetylation, are also involved.

The text in this review has been selective in discussing the field and the reader is advised to consult Tables 2 and 3 for a more comprehensive appreciation of the wider literature review.

## Conclusions and future perspectives

The results of our systematic literature assessment demonstrate a growing interest in the contribution of histone modifications in regulating the development of allergic disorders and, at the same time, provide evidence supporting this contribution. The role of histone modification is manifested at least at two levels. One involves the regulation of cells participating in the allergic inflammatory reaction, namely the inflammatory cells, T cells and macrophages, and the local tissue cells, such as (myo-) fibroblasts, which contribute to remodeling of airways. The other is the direct relationships between histone modifications and allergic phenotypes.

Furthermore, experimental observations of effects of histone marks modifying substances, e.g. HDACis or HMTis, suggest the potential application of histone epigenome editing in the treatment of allergies [35, 86–92]. Such therapies do not need to be simply restricted to histone-modifying enzyme inhibitors but may also include more targeted approaches based on e.g. CRISPR/dCas9 system [6, 92] or antisense molecules [6, 93–95]. Others include nutrients [71] or even bio-physical interventions [96]. Finally, also diagnostic/prognostic tools for allergic traits based on epigenetic patterns/signatures could possibly be developed in future, as suggested by several studies on DNA methylation [6, 97–99].

This review provides a systematic update of the current knowledge on the contribution of histone modifications to allergic inflammation and disorders.

## Abbreviations

*ADAM33*: ADAM metallopeptidase domain 33 (ADAM33) gene
ASMC: airway smooth muscle cell
BET (proteins): bromodomain and extra-terminal (proteins)
CB: cord blood
*CCL2*/*17*/*22*: C–C motif chemokine ligand 2/17/22 gene
*COX2*: cytochrome c oxidase subunit II (COX2) gene
*CXCL8*: C–X–C motif chemokine ligand 8 (CXCL8) gene
DOT1L (human KMT): DOT1-like (human KMT)
DUB: deubiquitinating enzyme
*DUSP1*: dual specificity phosphatase 1 (MAPK phosphatase 1; MKP-1) gene
*EGFR*: epidermal growth factor receptor gene
EZH2: enhancer of zeste 2 polycomb repressive complex 2 subunit
FCS: fetal calf serum
*GATA3*: GATA binding protein 3 (GATA3) gene
GNAT (family): GCN5-related *N*-acetyltransferase (family)
HAT: histone acetyltransferase
HDAC: histone deacetylase
HDACi: HDAC inhibitor
HDM: histone demethylase
HMT: histone methyltransferase
HMTi: HMT inhibitor
*IL6/9/13*: interleukin 6/9/13 (IL-6/-9/-13) gene
*IFNG*: interferon gamma (IFNγ) gene
IPF: idiopathic pulmonary fibrosis
JMJC (domain): JumonjiC (domain)
KMT: lysine methyltransferase
LSD/KDM1: (amine-oxidase type) lysine-specific demethylase
PRMT: arginine methyltransferase
PCAF: p300/CBP-associated factor
*PRKCZ*: protein kinase C zeta (PKCζ) gene
*PTGER2*: prostaglandin E receptor 2 (EP2) gene
p300: E1A binding protein p300
SAM: S-adenosyl-l-methionine
*STAT6*: signal transducer and activator of transcription 6 gene
*TBX21*: T-box 21 (TBX21) gene
TGF-β: transforming growth factor beta
Th (cell): helper T-cells/T-helper (cell)
*TP63*: tumor protein p63 gene
*VEGFA*: vascular endothelial growth factor A (VEGF) gene
